# Ionizing Radiation Increases Death Receptor 5 (DR5)-Mediated Cell Death, but Not Death Receptor 4 (DR4)-Mediated Cell Death in 3D Tumor Spheroids

**DOI:** 10.3390/ijms26104635

**Published:** 2025-05-13

**Authors:** Fengzhi Suo, Xinyu Zhou, Abel Soto-Gamez, Fleur B. Nijdam, Rita Setroikromo, Wim J. Quax

**Affiliations:** 1 Department of Chemical and Pharmaceutical Biology, Groningen Research Institute of Pharmacy, University of Groningen, Antonius Deusinglaan 1, 9713 AV Groningen, The Netherlands; f.suo@rug.nl (F.S.); r.setroikromo@rug.nl (R.S.); 2 Department of Radiation Oncology and Department of Biomedical Sciences, University Medical Center Groningen, Hanzeplein 1, 9713 GZ Groningen, The Netherlands

**Keywords:** TRAIL-induced apoptosis, death receptor (DR), 3D tumor spheroids, ionizing radiation

## Abstract

Tumor necrosis factor (TNF)-related apoptosis-inducing ligand (TRAIL) is a potential therapeutic for cancer patients due to its tumor specificity. However, TRAIL resistance in cancer cells limits its development in clinical trials. Given that ionizing radiation (IR) is an established method of inducing DNA damage for cancer during radiotherapy, we applied a combined treatment of IR and TRAIL. Our study shows that the combination treatment of IR and TRAIL promoted cell death due to IR upregulating both DR4/DR5 receptors on the surface of human lung carcinoma cell line H460 and human colon cancer cell line DLD-1 2D cells. However, when cultured as 3D spheroids, we observed that IR enhanced DR5-specific TRAIL-induced cell death but attenuated DR4-specific TRAIL-induced cell death. The immunohistochemical analysis of 3D cell spheroid sections indicates that it is due to a lack of DR4 overexpression by IR. Our findings elucidate a potential explanation for the failure of the combination treatment of radiotherapy with TRAIL in clinical trials. Additionally, our findings advocate the potential efficacy of employing DR5-specific TRAIL in combination with radiation as a promising therapeutic strategy.

## 1. Introduction

Tumor necrosis factor (TNF)-related apoptosis-inducing ligand (TRAIL) is described as selectively inducing cell apoptosis in cancer cells while sparing untransformed cells [1,2]. As a member of the TNF superfamily, TRAIL has five identified receptors, which all belong to the TNF receptor superfamily. TRAIL binds to death receptor 4 (DR4, also called TRAILR1) or death receptor 5 (DR5, also called TRAILR2) to initiate the TRAIL-induced extrinsic apoptosis signaling pathway [3], whereas decoy receptor 1 (DcR1, or TRAILR3), decoy receptor 2 (DcR2, or TRAILR4), and soluble receptor osteoprotegerin (OPG) attenuate apoptosis by competitive binding to TRAIL [4]. The assembly of TRAIL with DR4/DR5 recruits the Fas-associated death domain (FADD) and pro-caspase-8 [5]. The activated caspase-8 further cleaves pro-caspase-3 into caspase-3, which triggers cell apoptosis and DNA fragmentation [6,7]. TRAIL has been considered a promising anti-cancer therapeutic since its discovery. However, some tumor cells are resistant to TRAIL-induced apoptosis, limiting TRAIL development in cancer treatment [8]. Therefore, more research has focused on alternative strategies for overcoming TRAIL resistance in tumor cells, such as inducing the upregulation of DR4 or DR5, the downregulation of decoy receptors, or the inhibition of c-FLIP, XIAP, or Bcl-2 family members [9,10,11].

Although some tumor cells are sensitive to TRAIL-induced apoptosis in vitro, they show resistance in animal assays or clinical trials [12]. This suggests that solid tumors possess a different sensitivity to TRAIL compared with their corresponding 2D cells. The formation of three-dimensional (3D) spheroids is a potential technology to mimic solid tumors in vitro and to achieve better precision in drug discovery. In comparison to conventional adherent 2D cells, 3D tumor spheroids better simulate cellular interactions and architectures of solid tumors as well as their characteristic properties, such as hypoxia and drug resistance [13,14]. Recently, Stöhr et al. found that the expression level of death receptors on the cell surface differs based on the location of tumor cells within the spheroid. The intermediate layer is formed by cells with low or absent expression of death receptors, protecting cancer cells from TRAIL-induced cell death [15]. These findings may explain the failure of TRAIL therapy in clinical trials. The findings also aroused our interest in investigating the differential expression of death receptors in 2D tumor cells and 3D spheroids.

At the same time, the combination of TRAIL and drugs that cause DNA damage is proving effective. Research on TRAIL in clinical trials is usually performed as a combination treatment with chemotherapy or radiotherapy. Radiotherapy is still one of the most common modalities, using high doses of ionizing radiation to limit or kill tumor cells [16]. Ionizing radiation generates reactive oxygen species (ROS), which are chemically reactive molecules containing oxygen, resulting in DNA single- and double-strand breaks, irregular gene expression, and numerous cell signaling pathways related to apoptosis, necrosis, and proliferation [17,18]. It has been reported that ionizing radiation stimulates DR4 and DR5 expression in multiple tumor cell lines in vitro [19,20,21,22,23], as well as decoy receptors [24,25], but this cannot explain the limitations of the combination of radiotherapy with TRAIL in clinical trials. However, these studies are performed in 2D cells, and there are limited studies on the effects on 3D cells and tumors in vivo. Meanwhile, there are no positive reports for the combination of radiotherapy and TRAIL in clinical trials (ClinicalTrials.gov ID: NCT01017822, NCT01088347).

It is important to explore the sensitivity of different biomimetic tissue models to receptor-specific TRAIL variants to understand the role of death receptors in TRAIL resistance. Therefore, to uncover the difference between adherent 2D cells and 3D spheroids, we analyzed their cell surface death receptor expression and their sensitivity to receptor-specific TRAIL variants. Then, the influence of ionizing radiation on death receptor expression was measured, suggesting that ionizing radiation increases DR5 expression in both biomimetic tissue models, but not DR4 expression in 3D spheroids. Finally, the results show that ionizing radiation has a synergistic effect with the DR5-specific TRAIL variant, but not with the DR4-specific TRAIL variant.

## 2. Results

### 2.1. TRAIL-Induced Cell Death in 2D Monolayer H460 and DLD-1 Cells

**Figure 1:**
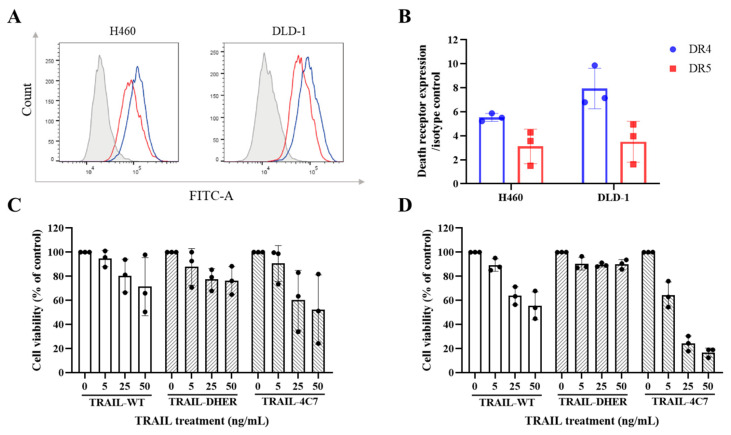
TRAIL variants induce cell death in 2D monolayer H460 and DLD-1 cells. (**A**) Histograms, measured by flow cytometry, display the expression of DR4 and DR5 on the cell surface of 2D monolayer H460 and DLD-1 cells (Gray peak: isotype; Blue: DR4; Red: DR5). (**B**) The median fluorescence intensity (MFI) ratio relative to that of the IgG isotype. (**C**,**D**) As determined by MTS assay, recombinant TRAIL and TRAIL variants reduce the viability of H460 and DLD-1 cells compared to untreated control (0 ng/mL). Data shown are mean ± SD from three independent experiments performed in triplicate.

In our study, we utilized two cancer cell lines, namely H460 and DLD-1, representing non-small cell lung cancer and colon cancer, respectively. First, we assessed the expression status of DR4 and DR5 on the surface of H460 and DLD-1 monolayer cells. As shown in Figure 1A, the histogram curves for DR4 and DR5 in both cell lines exhibited a shift to the right, relative to the corresponding isotype control, indicating increased expression. The relative expression values of DR4 compared to the isotype were 5.5 and 7.1, respectively, while the expression values of DR5 were 3.9 and 4.4, respectively (Figure 1B). Subsequently, TRAIL-WT and its variants, DR5-specific TRAIL-DHER and DR4-specific TRAIL-4C7, were added to monolayer H460 and DLD-1 cells with concentrations ranging from 5 to 50 ng/mL (Figure 1C,D). TRAIL-WT, TRAIL-DHER, and TRAIL-4C7 all reduced the cell viability of H460 cells, with TRAIL-4C7 demonstrating a more pronounced effect and 48% reduction at 50 ng/mL. At a concentration of 25 ng/mL, TRAIL-DHER induced a 20% reduction in viability, with no increase in reduction observed upon escalation to 50 ng/mL. DLD-1 cells are more sensitive to DR4-specific TRAIL-4C7 and a bit more resistant to DR5-specific TRAIL-DHER compared to H460. TRAIL-4C7 resulted in more than 80% cell viability reduction at 50 ng/mL, whereas TRAIL-DHER only reduced cell viability by 10%.

### 2.2. IR Enhances TRAIL-Induced Apoptosis in 2D Monolayer Cancer Cells

**Figure 2:**
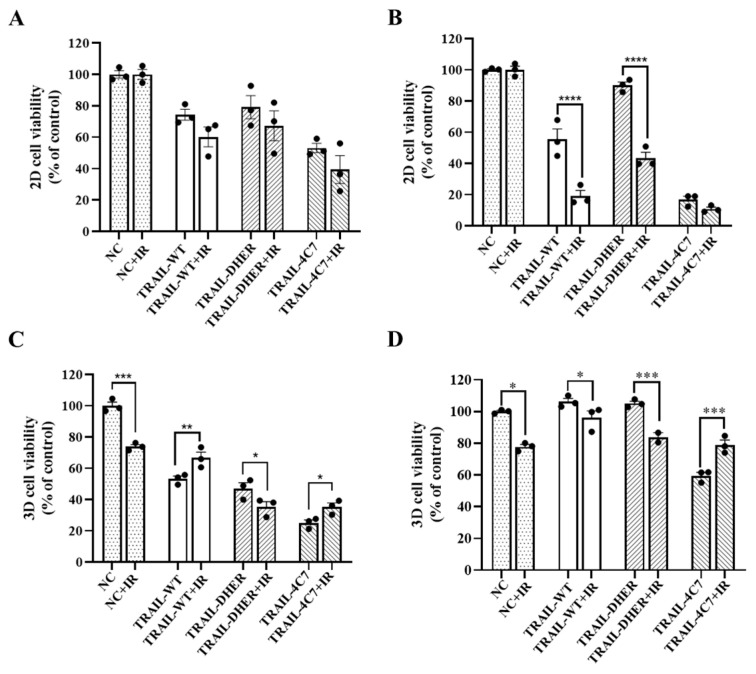
Differential response of 2D vs. 3D cells to ionizing radiation and TRAIL variant combinations. (**A**,**B**) Cell viability of 2D H460 and DLD = 1 cells treated by TRAIL variants and ionizing, expressed as a percentage of the non-treated control (NC). (**C**,**D**) Cell viability of 3D H460 and DLD = 1 cells treated by TRAIL variants and ionizing, expressed as a percentage of the non-treated control (NC). Cell viability was measured using an MTS assay. Data shown are mean ± SD from three independent experiments performed in triplicate. * 0.01 < *p* ≤ 0.05, ** 0.001 < *p* ≤ 0.01, *** 0.0001 < *p* ≤ 0.001, **** *p* ≤ 0.0001.

Given the well-established role of IR in inducing DNA damage as a conventional therapeutic approach in cancer treatment, we implemented a combined treatment involving TRAIL and IR for H460 and DLD-1 cell lines. As depicted in Figure 2A, treatment with TRAIL-WT, TRAIL-DHER, and TRAIL-4C7 decreased cell viability by 26%, 47%, and 21%, respectively, in H460 cells. Furthermore, the additional administration of IR resulted in a greater decrease in cell viability of 40%, 61%, and 33% in the respective groups. In Figure 2B, the synergistic effect of IR on TRAIL is more evident in DLD-1 cells: TRAIL-WT, TRAIL-DHER, and TRAIL-4C7 treatment decreased cell viability by 45%, 89%, and 10%, respectively, whereas additional administration of IR resulted in a greater decrease in cell viability of 81%, 89%, and 57%, respectively.

### 2.3. IR Inhibits DR4-Mediated Cell Death in 3D Cancer Spheroids

We then used cell spheroids to test this combined approach. The structure and interactions of tumor cells in a three-dimensional setting are modeled more accurately by this method. For this, tumor cells were first cultivated in ultra-low attachment 96-well plates and cultured for 5 days to form spheroids. Interestingly, H460 cells, which were more resistant to TRAIL in the 2D system compared with DLD-1 cells, are more sensitive than DLD-1 as 3D spheroids (Figure 2C,D). What is more, although both cells in the 2D system showed a synergistic effect of IR with TRAIL treatment, this was not always the case in the 3D system. We noticed that IR inhibited TRAIL-WT-induced cell death in H460 cells, while enhancing TRAIL-WT-induced cell death in DLD-1 cells. Moreover, there was a synergistic effect of IR with TRAIL-DHER on cell death: IR augmented cell death by an additional 12% in H460 cells and 21% in DLD-1 cells. However, IR antagonized the effects of TRAIL-4C7-induced cell death, resulting in a decrease of 10% cell death in H460 cells and 19% in DLD-1 cells.

**Figure 3:**
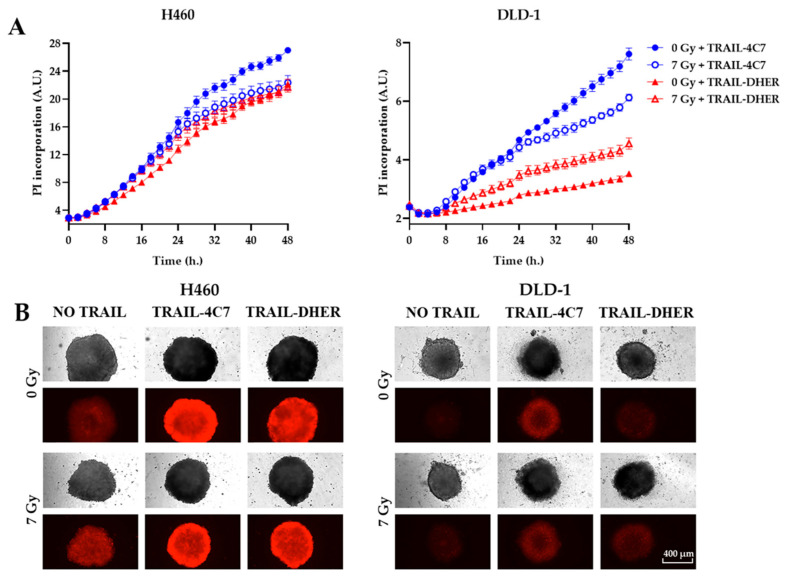
Ionizing radiation differentially impacts cell death induced by TRAIL variants TRAIL-4C7 and TRAIL-DHER in H460 and DLD-1 spheroids. (**A**) The spheroids of H460 and DLD-1 cells were pre-treated with/without ionizing irradiation (7 Gy) and cultured for 24 h, followed by 50 ng/mL of TRAIL-4C7 or TRAIL-DHER for 24 h. Time-resolved assays were performed using propidium iodide fluorescent intensity as a measure of cell death. (**B**) Representative images of the bright field and fluorescence images of spheroids after TRAIL treatment are shown. Data shown are mean ± SD from three independent experiments performed in triplicate.

To further validate the cell death results obtained through cell viability detection, we used propidium iodide (PI), a DNA-binding fluorescent dye used to identify dead cells) staining. A time-resolved assay was conducted to intuitively investigate the effects of the combined treatment of ionizing radiation with receptor-specific TRAIL variants. In both H460 and DLD-1 3D spheroids, 7 Gy irradiation enhanced the efficacy of TRAIL-DHER in inducing cell death (as indicated by PI intensity) compared to the unirradiated group, while it inhibited the effectiveness of TRAIL-4C7 (Figure 3A). This outcome was time-dependent, with the difference being more pronounced in DLD-1 cells. Among all tested groups, individual TRAIL-4C7 treatment showed the highest cytotoxicity. As shown in Figure 3B, irradiation treatment restricted the growth of tumor spheroids in both H460 and DLD-1. However, under TRAIL-4C7 treatment, the PI fluorescence of the 0 Gy group was higher than that of the 7 Gy group in both cell lines, whereas the TRAIL-DHER group showed the opposite trend. In summary, irradiation treatment led to contrasting effects on TRAIL-4C7 and TRAIL-DHER-induced cell death in 3D spheroids.

### 2.4. IR Upregulates the Expression of DR5, but Not DR4 in 3D Spheroids

**Figure 4:**
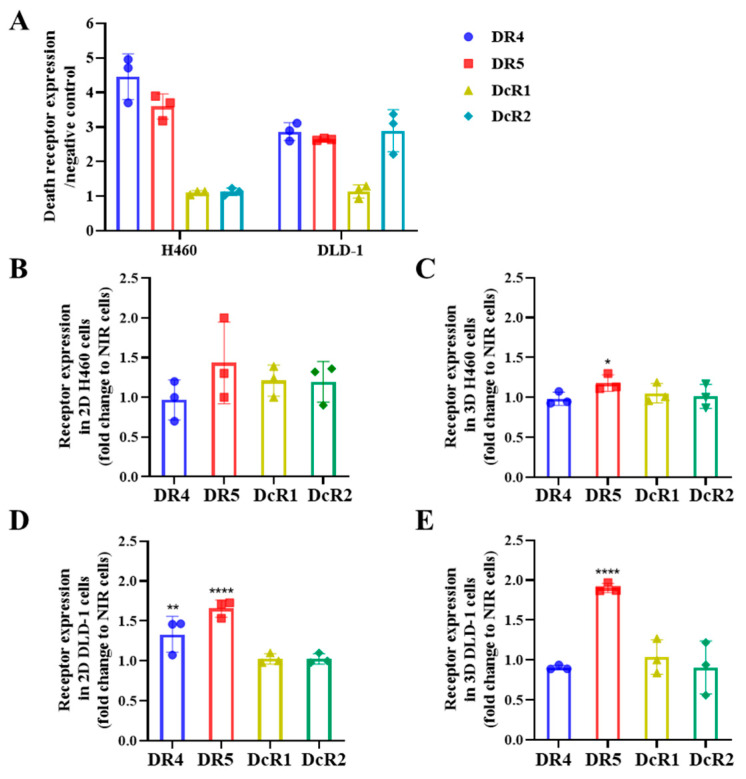
Receptor expression of H460 and DLD-1 by flow cytometry. (**A**) DR4, DR5, DcR1, and DcR2 expression on the cell surface of the 3D spheroids H460 and DLD-1. (**B**,**C**) Fold changes in DR4, DR5, DcR1, and DcR2 expression in 2D and 3D H460 cells before and after radiation exposure. (**D**,**E**) Fold changes in DR4, DR5, DcR1, and DcR2 expression in 2D and 3D DLD-1 cells before and after radiation exposure. In the bar charts, all data are normalized to the negative control. Data shown are mean ± SD from three independent experiments. * 0.01 < *p* ≤ 0.05, ** 0.001 < *p* ≤ 0.01, **** *p* ≤ 0.0001. Gating strategy and receptor expression histogram analysis in 3D tumor models are described in detail in Supplementary Figures S1 and S2.

Stöhr et al. reported that the formation of cell spheroids lead to a reduction of DR4/DR5 expression [15]. Radiation-induced modulation of death receptor expression could influence the efficacy of TRAIL variants. Based on this, we investigated the receptor expression in irradiated 3D spheroids, including potential alterations in decoy receptors (DcR1/DcR2) that might affect apoptosis outcomes. Single cells from the 3D spheroids were isolated through trypsinization, and the expression of death receptors was analyzed using flow cytometry. As shown in Figure 4A, we observed lower surface expression of both DR4 (MFI ratios: H460 = 4.4, DLD-1 = 2.7) and DR5 (MFI ratios: H460 = 3.6, DLD-1 = 2.6) compared to 2D cultures, as seen in Figure 1A, consistent with previous Daniela’s report of spheroid-associated death receptor downregulation. Furthermore, the expression of both DcR1 and DcR2 was observed in 3D spheroids, with markedly low levels of DcR1.

Interestingly, radiation induced differential expression patterns between DR4 and DR5 in the 2D and 3D systems. As we can see in Figure 4B,D, IR upregulated DR5 expression while inducing moderate regulation of DR4 expression (H460: n.s.; DLD-1: *p* < 0.01) in 2D cells. Decoy receptor expression showed minimal changes after irradiation. In 3D spheroids (Figure 4C,E), radiation enhanced DR5 while slightly suppressing DR4 levels. Decoy receptors also showed minimal changes after irradiation, which suggests that decoy receptors play a limited role in modulating the anti-tumor responses.

### 2.5. Ionizing Radiation Only Inhibits the Upregulation of DR4 in 3D Cells

**Figure 5:**
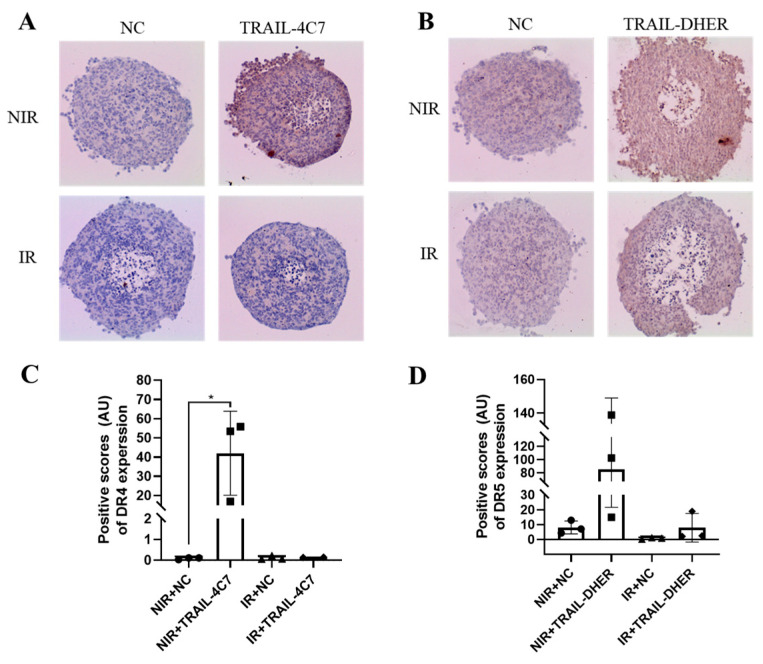
DR4 and DR5 expression in TRAIL and IR combined treated 3D DLD-1 spheroids. Spheroids are sliced into 4 µm thick sections. The sections are then stained with (**A**) DR4 or (**B**) DR5 antibodies for immunohistochemistry analysis. DAB brown precipitate is used to detect the presence and distribution of the protein of interest. (**C**,**D**) Quantification of DAB staining. Data shown are mean ± SD from three experiments. * 0.01 < *p* ≤ 0.05.

To investigate death receptor expression directly in the 3D spheroids, we performed immunohistochemical (IHC) analysis, a technique that detects and visualizes specific proteins within tissue sections. After the spheroids reached a diameter of around 500 µm after 7 days in culture, they were sliced and stained for DR4 (Figure 5A) and DR5 (Figure 5B).

We noticed that single treatment of TRAIL-4C7 or TRAIL-DHER leads to a notable increase in DR4 or DR5 expression, where DR4 is particularly in the outermost spheroid layer, and DR5 is uniformly in the spheroids. However, IR treatment abolished TRAIL-4C7-induced DR4 upregulation in both the outer layer and the intermediate layer of the spheroids. In contrast, IR did not change the upregulation of DR5 by TRAIL-DHER. The quantized data are presented in Figure 5C,D.

In summary, the differential regulation of death receptors by IR provides mechanistic insight into tumor cell sensitivity after TRAIL variant treatment.

## 3. Discussion

Targeted therapy, which targets specific molecules to inhibit tumor progression and metastasis, has become a hot topic and a research emphasis due to its tumor cell specificity and reduced side effects on healthy cells [26,27]. Among all candidates, TRAIL has drawn attention since its discovery in 1995, given that it specifically induces tumor cell death [28]. The resistance of some cancer cells to TRAIL-induced apoptosis results in the limitation of its use as an effective therapy in cancer treatment. Since the development of X-rays in 1895, ionizing radiation has been used for cancer treatment up to today [29,30]. Our research group has dedicated substantial efforts to elucidating the mechanisms underlying drug resistance to TRAIL and has subsequently formulated strategies aimed at overcoming TRAIL resistance based on these findings [8].

We tested the apoptotic efficacy of TRAIL and its receptor-specific variants in DLD-1 and H460 as 2D cell monolayers. Both cell lines were sensitive to the DR4-specific TRAIL variant (TRAIL-4C7) compared to the DR5-specific variant (TRAIL-DHER). After combined treatment with IR, we observed a synergistic effect of IR with TRAIL and its variants to induce 2D cell death. Other researchers also found that IR acted synergistically on DR4/DR5-mediated apoptosis, which appears to be P53-dependent [19,21,22,31]. Importantly, although the P53-dependent induction of decoy receptors in response to chemotoxic agents may compromise wild-type TRAIL efficacy in monolayer cultures, this phenomenon diverges in our 3D spheroid models [24,25]. In our experimental system, DcR surface expression in 3D spheroids remained unaffected by IR. Moreover, the receptor-specific TRAIL used in this study maintains high binding affinities for individual death receptors [32].

To simulate the in vivo environment of solid tumors more accurately, we cultured 3D cell spheroids to test the combination treatment of IR and TRAIL. We noticed that IR promotes cell death induced by TRAIL-DHER in our 3D tumor spheroids, yet it exerts opposing effects on the efficacy of TRAIL-4C7. Zerp et al. also found that treatment with IR and a pan-TRAIL receptor agonist (APG-880) enhanced cytotoxicity in patient-derived colorectal carcinoma 3D organoids [33]. In their research, Marini et al. used HGS-ETR1 and HGS-ETR2, which are monoclonal antibodies specific to DR4 or DR5, respectively. They found a synergistical anti-tumor effect of IR with both ETR1 and ETR2 in vitro. However, the cell lines used in that study expressed low to non-measurable levels of DR4 [34].

We investigated whether differential sensitivity to IR/TRAIL treatment correlated with DR4/DR5 expression. The formation of 3D DLD-1 spheroids resulted in 2.6-fold (7.1/2.7) and 1.7-fold (4.4/2.6) lower levels of DR4 and DR5 than in 2D cells. The same findings were reported by Stöhr et al. [15]. After IR treatment, DR4 and DR5 expression increased on the surface of 2D cells, whereas the upregulation of DR4 was not observed in 3D cells. Combination treatment with IR suppressed the elevation of DR4, while DR5 expression remained high. DR5 was reported to be upregulated in response to IR [34]. A high level of DR5 expression was found two days post-irradiation, which led to the suppression of tumor growth rates in xenograft models derived from both MKN45 and MKN28 cells in a p53-independent manner [35]. Di Pietro et al. identified that radiation sensitized 2D erythroleukemic cells to TRAIL-mediated cytotoxicity by selectively upregulating DR4 expression [36]. However, another study revealed that the expression of DR4 in breast cancer exhibited no significant alterations [19]. Hence, there exists some controversy regarding the regulation of death receptor expression, with notable variations observed across different cell lines [37].

Previous research by our group has highlighted the importance of epigenetic regulation in modulating the expression of DR4 and DR5 expression [38,39], and IR can alter global acetylation and methylation levels in cells [40,41,42]. In our previous research, we observed that treatment with RGFP966, a histone deacetylase (HDAC) inhibitor, led to an increase in DR4 protein expression in DLD-1 cells, whereas DR5 expression remained unaffected [39]. Similarly, treatment with another HDAC inhibitor, MS-275, resulted in the upregulation of DR4 protein levels without affecting DR5 protein levels [43]. Additionally, the transcript expression level of DR4 was observed to display a negative correlation with methylation status, which can be modulated by radiation [44,45]. These findings suggest that the expression level of DR4 is more susceptible to radiation-induced dynamic histone modifications compared to DR5.

Our study provides mechanistic insights into the different roles of DR4 and DR5 activation under IR treatment using trimeric TRAIL variants. Whereas our receptor-selective variants mimic genuine receptor interactions, we acknowledge that additional validation with receptor knockout models or engineered antibodies could give additional insight. Future studies exploring these approaches might strengthen the impact of our study. Despite these limitations, our findings highlight the therapeutic potential of targeting DR5 with a specific trimeric ligand.

## 4. Materials and Methods

### 4.1. Cell Lines and Reagents

Human lung carcinoma cell line H460 and human colon cancer cell line DLD-1 were obtained from American Type Culture Collection (ATCC, Manassas, WV, USA). The cells were cultured in RPMI-1640 supplemented with 10% fetal bovine serum (FBS), 100 units/mL penicillin, and 100 µg/mL streptomycin. All the cells were cultured in a humidified incubator at 37 °C and 5% CO_2_. The recombinant human TRAIL (rhTRAIL) wild type (TRAIL-WT), DR4-specific TRAIL variant (TRAIL-4C7), and DR5-specific TRAIL variant (TRAIL-DHER) were produced as described previously [46,47]. For irradiation therapy, cells were sham-irradiated (0 Gy) or irradiated with 7 Gy using an IBL 637 Cesium-37 gamma ray machine (dose rate 0.0082 Gy/s, then incubated for 24 h before the following treatments.

### 4.2. Cell Viability Assay for 2D

For 2D cells, cell viability was determined using an MTS assay, which is a colorimetric method for evaluating cell proliferation (Promega Corporation, Madison, WI, USA). The cells were seeded in triplicate in 96-well plates at a density of 5000 cells/well in 100 μL medium for 24 h. Then, the cells were treated with/without 7 Gy of photon irradiation and incubated for 24 h, followed by recombinant human TRAIL-WT, TRAIL-4C7, or TRAIL-DHER. After 24 h of treatment, the cells were incubated with 20 μL MTS reagent according to the manufacturer’s instructions. Cell viability was recorded by measuring absorption at 490 nm on a POLARstar Omega microplate reader (BMG LABTECH GmbH, Ortenberg, Germany).

### 4.3. D Spheroid Construction and Cell Viability Assay

The cells were seeded at a density of 2000 cells/well in ultra-low attachment 96-well plates (Corning Incorporated, Corning, NY, USA). The plates were centrifuged at 1000 rpm for 10 min and incubated for 3 days. After the 3D spheroid formation, they were irradiated with 7 Gy of photon irradiation followed by recombinant human TRAIL-WT, TRAIL-4C7, or TRAIL-DHER treatment. After 24 h of treatment, an equal volume of CellTiter-Glo^®^ 3D Reagent (Promega Corporation, Madison, WI, USA) was added to each well according to the manufacturer’s instructions. After 30 min incubation at room temperature, all the contents were transferred into white-walled 96-well plates and the luminescence values were recorded using a Synergy™ H1 plate reader (BioTek Instruments, Winooski, VT, USA).

### 4.4. Incucyte ^®^ ZOOM Time-Resolved Assays

The cells were maintained at 37 °C with 5% CO_2_ and imaged using an IncuCyte^®^ ZOOM (Essen BioScience, Newark, UK) with a 4× magnification. The cells were irradiated with 7 Gy. Then, for cell death determination, recombinant human TRAIL-WT, TRAIL-4C7, or TRAIL-DHER with 2.5 μg/mL of propidium iodide (PI) were added into each well and the fluorescent signal was recorded by IncuCyte^®^ ZOOM every 2 h.

### 4.5. Flow Cytometry of Death Receptor Expression

The cells were seeded in 6-well plates at a density of 300,000 cells/well overnight. After 7 Gy irradiation and 24 h incubation, the cells were harvested and washed with FACS buffer (PBS with 2% FBS). For 3D cells, all the spheroids were collected and trypsinized into single cells and washed with FACS buffer. Anti-DR4 antibody (Abcam, Cambridge, UK), anti-DR5 antibody (Exbio, Praha, Czech Republic), anti-DcR1 (R&D system, Minneapolis, MN, USA), and anti-DcR2 (R&D system, Minneapolis, MN, USA) were incubated with the cells for 1 h at 4 °C. Subsequently, the cells were washed and incubated with Fluorescein (FITC)-conjugated donkey anti-mouse IgG (Jackson ImmunoResearch, Cambridge, UK) for 1 h at 4 °C. Isotype control staining was performed with mouse IgG (Dako, Glostrup, Denmark). Death receptor expression was determined using the NovoCyte Quanteon system (ACEA Biosciences, San Diego, CA, USA).

### 4.6. Spheroid Embedding and Sections

Spheroids were collected in a 1.5 mL reaction tube, washed with PBS twice, and fixed with formaldehyde with Ponceau S (4% formaldehyde: Ponceau S = 14:1) for 20 min at room temperature. Ponceau S was used to track the spheroids. After removal of formaldehyde, the spheroids were treated with ethanol solutions (70%, 90%, and 95% for 5 min each) for dehydration, followed by 100% ethanol (3 times for 5 min each), and 100% xylene (3 times for 5 min each) for clearing. The spheroids were transferred to liquid paraffin (VWR International, Radnor, PA, USA) for 15 min and moved to a cold plate for rapid solidification. Samples were cut into 4 μm slices (Leika, Wetzlar, Germany).

### 4.7. Immunohistochemistry for 3D Spheroid Sections

The sections were treated with xylene three times for 5 min each, followed by 100%, 100%, 95%, and 70% ethanol solutions for 3 min each. After washing again with PBS, the sections were incubated with retrieval buffer and heated to 100 °C for 25 min. Until the buffer cooled to room temperature, the sections were rinsed with PBS thrice for 5 min and incubated with 0.3% H202 TBS for 15 min. After washing, 10% BSA at 37 °C was used for blocking for 1 h. Primary DR4 or DR5 antibodies (Abcam, Cambridge, UK) were added to the sections overnight at 4 °C. After washing, secondary antibodies were added and incubated for 1 h at room temperature. Freshly made DAB solution was added to the slides according to the manufacturer’s instructions (Sigma-Aldrich, St. Louis, MO, USA). Cell nuclei were stained with hematoxylin solution (Merck, Darmstadt, Germany). Afterwards, the sections were treated with ethanol solutions (75%, 85%, 95%, 100%, and 100% for 3 min each) and incubated with xylene thrice for 5 min. The stained sections were mounted using DPX Mountant (Sigma-Aldrich, St. Louis, MO, USA). Data quantification was performed using Image J Version 1.54p (National Institutes of Health, Bethesda, MD, USA).

### 4.8. Statistical Analysis

Data were presented as mean ± standard deviation (SD) from three independent experiments. *p*-Values were analyzed using multiple comparisons test in GraphPad Prism version 8.0 (GraphPad Software, San Diego, CA, USA). * 0.01 < *p* ≤ 0.05, ** 0.001 < *p* ≤ 0.01, *** 0.0001 < *p* ≤ 0.001, **** *p* ≤ 0.0001. All flow cytometry data were analyzed using FlowJo v10.6 (FlowJo, LCC, Ashland, OR, USA).

## 5. Conclusions

This study showed that IR promoted the expression of DR4 and DR5 receptors on the surface of 2D cell monolayers, thereby enabling TRAIL-induced cell death in H460 and DLD-1 cells. However, in 3D spheroids, IR promoted DR5-mediated cell death while inhibiting DR4-mediated cell death.

Hence, we advocate the following considerations: (1) The suboptimal efficacy of TRAIL on solid tumors may stem from lower expression levels of DR4/DR5 in solid tumors compared to 2D cultures. Therefore, 3D models such as cell spheroids or organoids should be conducted to better mimic in vivo conditions. (2) Further investigation into the application of targeting DR5 in combination with IR therapy holds significant research value. Specifically, targeting a single receptor is essential to enhancing therapeutic efficacy and thereby achieving precise cancer treatment.

## Data Availability

The data that support the findings of this study are available from the corresponding author (W.J.Q.), upon reasonable request.

## Supplementary Materials

The following supporting information can be downloaded at https://www.mdpi.com/article/10.3390/ijms26104635/s1.
