# Supplementary material for: Ionizing Radiation Increases Death Receptor 5 (DR5)-Mediated Cell Death, but Not Death Receptor 4 (DR4)-Mediated Cell Death in 3D Tumor Spheroids
Source: Int J Mol Sci. 2025 May 13;26(10):4635. doi: 10.3390/ijms26104635 (PMC12110968; doi:10.3390/ijms26104635)
Supplement: Supplementary file 1 [file ijms-26-04635-s001.zip › ijms-3608305-supplementary.pdf]

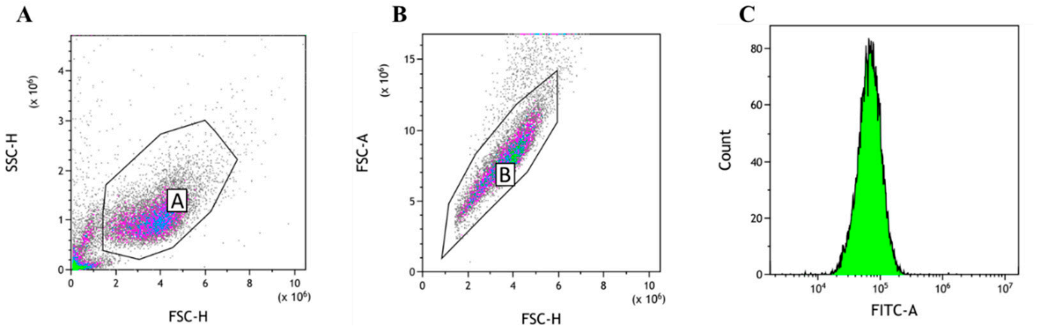

Supplementary Figure S1: Gating strategy for analysis of death receptor expression by flow cytometry. (A) Gate A was applied to identify viable cells based on forward scatter (FSC) and side scatter (SSC) characteristics. **(B)** Gate B was subsequently used to exclude cell aggregates and select single-cell populations. **(C)** FITC fluorescence histogram demonstrating death receptor expression profiles of cells was generated through Gates B.

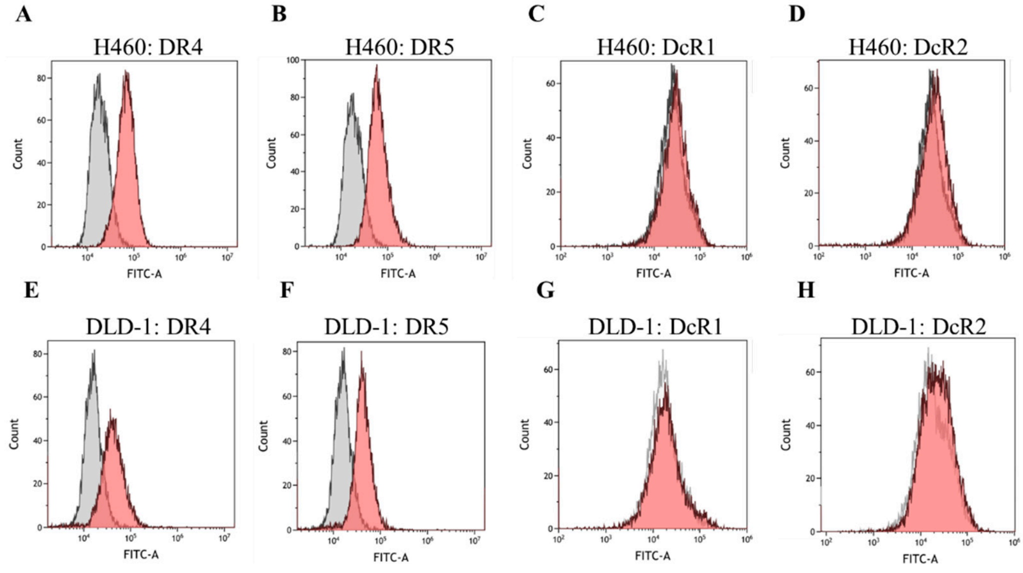

Supplementary Figure S2: Flow cytometry histograms of receptor expression in 3D-cultured H460 and DLD-1 cells. (A-D) DR4, DR5, DCR1, and DCR2 expression levels in H460 cells versus negative controls (secondary antibody-only staining). **(E-H)** DR4, DR5, DCR1, and DCR2 expression levels in DLD-1 cells versus negative controls (secondary antibody-only staining). Red histograms show receptor staining, while grey-shaded areas represent negative controls.
